# Molecular mechanisms underlying leaf development, morphological diversification, and beyond

**DOI:** 10.1093/plcell/koac118

**Published:** 2022-04-20

**Authors:** Hokuto Nakayama, Aaron R Leichty, Neelima R Sinha

**Affiliations:** Graduate School of Science, Department of Biological Sciences, The University of Tokyo, Tokyo 113-0033, Japan; Department of Plant Biology, University of California Davis, Davis, California 95616, USA; Department of Plant Biology, University of California Davis, Davis, California 95616, USA

## Abstract

The basic mechanisms of leaf development have been revealed through a combination of genetics and intense analyses in select model species. The genetic basis for diversity in leaf morphology seen in nature is also being unraveled through recent advances in techniques and technologies related to genomics and transcriptomics, which have had a major impact on these comparative studies. However, this has led to the emergence of new unresolved questions about the mechanisms that generate the diversity of leaf form. Here, we provide a review of the current knowledge of the fundamental molecular genetic mechanisms underlying leaf development with an emphasis on natural variation and conserved gene regulatory networks involved in leaf development. Beyond that, we discuss open questions/enigmas in the area of leaf development, how recent technologies can best be deployed to generate a unified understanding of leaf diversity and its evolution, and what untapped fields lie ahead.

## Teaser

A review of the current knowledge of leaf developmental genetics with an emphasis on natural variation and conserved gene regulatory networks involved in leaf development.

## Introduction

The variation in form seen in nature is astonishing and has fascinated biologists for centuries. How these varied forms arose in ontogeny and through evolutionary time has been the subject of intense study. Comparative morphology always argued for similarity of structures, by implication from shared descent. However, an understanding of the mechanistic basis of morphological variation required the theory of inheritance and the formulation of Mendel’s laws introduced in a paper published in 1866: “*Versuche über Pflanzen-Hybriden.*” With the discovery of DNA and the formulation of the central dogma of molecular biology, development and morphological diversity came to be understood as the readout of genes and their variations.

**Figure 1:**
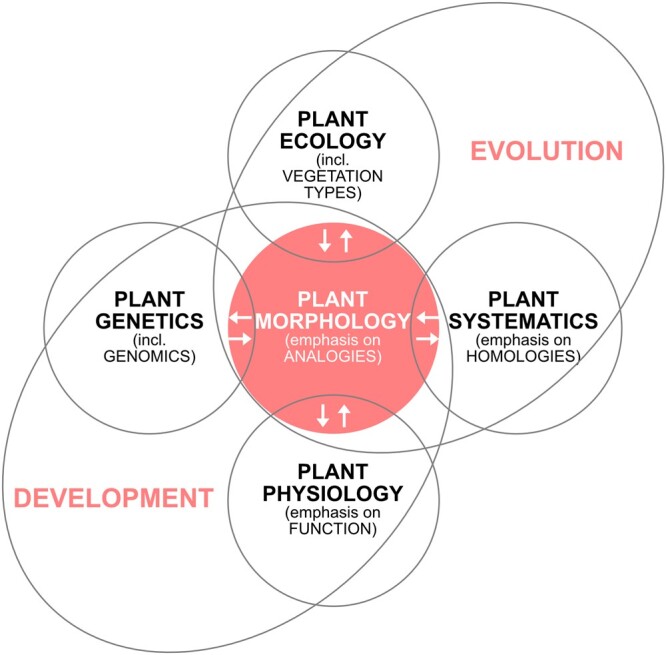
Diversity of form is central to the field of Evo-Devo. The analysis of leaf morphology and its convergence across taxa has been a central theme of plant morphology since Goethe (Kaplan, 2001). Over 200 years later, the field of Evo-Devo, with its emphasis on comparative genetics, has greatly informed our understanding of the mechanisms that underlie these convergences in form and function. New tools applied to new and old model systems are clarifying the hidden diversity of developmental mechanisms. Figure modified from Kaplan (2001).

The diversity of leaf forms seen in nature was a key inspiration in early analyses of plant morphology (Von Goethe, 1790). Goethe’s hypothesis that the leaf is the basic building block of all vegetative organs arising from the meristem (i.e. an archetype) was probably one of the first formal evolutionary developmental hypotheses. This approach of identifying principles that “transcend…systematic boundaries” has been central to the field of plant morphology and the newer field of evolutionary developmental biology (Evo-Devo; Figure 1; Kaplan, 2001). Indeed, Evo-Devo over the past three decades has been largely dominated by comparative gene studies using a phylogenetic framework, yet many of the findings have resulted in predictable genetic mechanisms for explaining similarities in morphology, independent of evolutionary history. Importantly, these analogies in developmental genetic mechanisms provide a framework for hypothesis testing for the extensive leaf diversity yet to be examined at the molecular level.

We begin this review by highlighting the fundamental molecular genetic mechanisms underlying leaf development derived from model systems, highlighting important recent additions. We then discuss how these mechanisms have been utilized in the generation of leaf diversity within plant lineages. We also discuss how many of the fundamental genetic regulatory pathways transcend many phylogenetic boundaries in the plant lineage and unify common morphologies and their derivations. Finally, we emphasize how placing traditional Evo-Devo systems into an ecological context aids in this understanding and how some cutting edge genomics technologies can best be deployed to generate a unified understanding of leaf diversity and its evolution.

## Basic mechanism of simple leaf development

**Figure 2:**
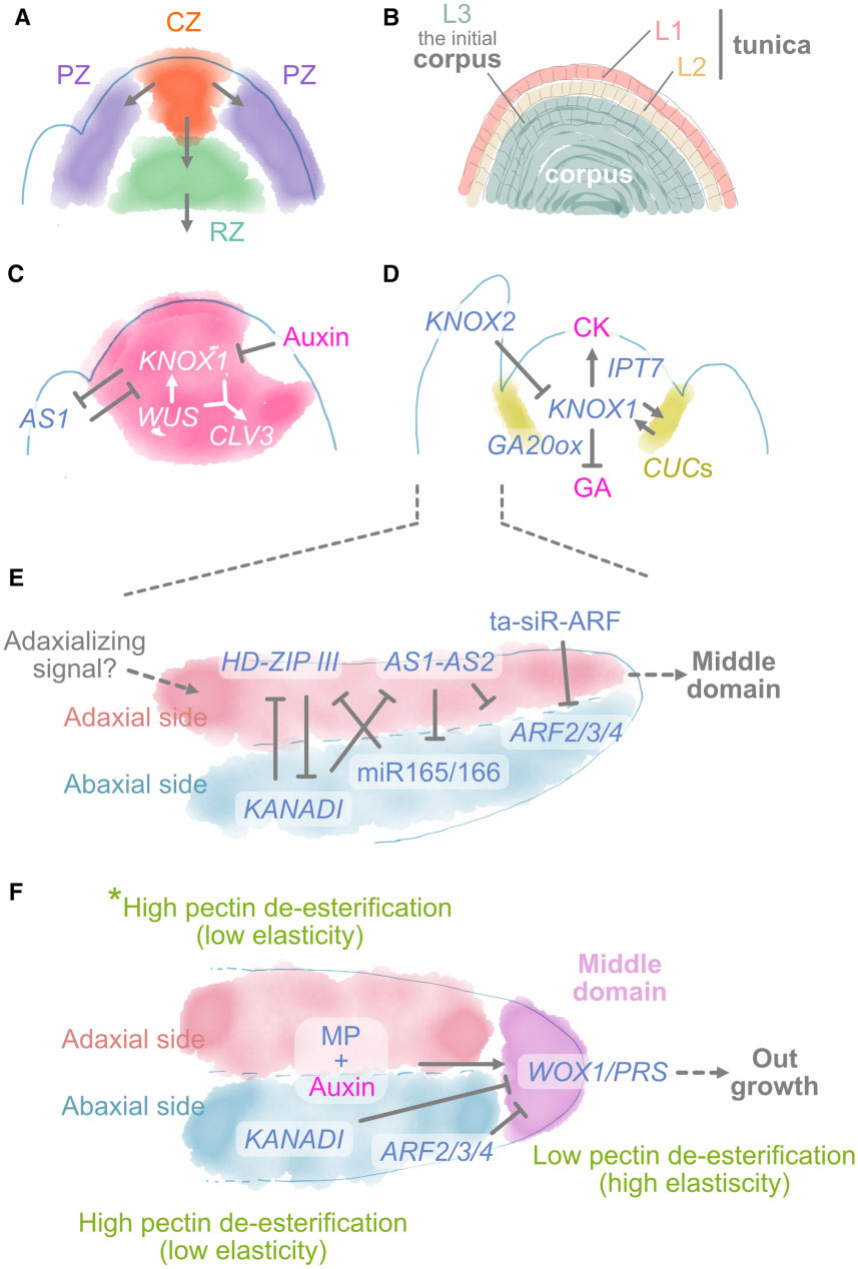
Schematic model of development in shoot apical meristem and leaf primordia. A, Cytohistological zonation of the SAM. B, The tunica-corpus model. C and D, Overview of the regulatory model for SAM maintenance and organogenesis; leaf initiation (C) and a genetic network of *KNOX1* regulation (D). E, A genetic network for leaf ad–ab polarity specification. F, A genetic network connecting the ad, middle, and ab domains. *Note that the ad side initially has low pectin de-esterification, followed by high pectin de-esterification.

Unlike cotyledons, which are produced embryonically, most plants produce leaves via post-embryonic activity at the shoot apical meristem (SAM). The SAM is divided into three regions: the central zone (CZ) containing pluripotent stem cells, the peripheral zone (PZ) where organ primordia emerge, and the rib zone (RZ), which gives rise to stem tissues (Figure 2A). Additionally, the meristem can be subdivided based on the cell layers that span these zones, often with a two-layer tunica (L1 and L2) and several layers of “corpus” (the initial layer is designated as L3; Figure 2B), which are distinguished by their direction of cell division (Schmidt, 1924). The CZ and PZ are mainly involved in leaf development. Stem cells in the CZ provide undifferentiated cells that serve as founder cells. Subsequently, the founder cells differentiate into lateral organs, such as leaves, in the PZ (Figure 2A).

This lateral organ initiation activity is integrally connected to the phytohormone auxin. Although auxin has various functions in post-embryonic leaf development, it also plays a role in leaf primordium initiation (Kalve et al., 2014). Specifically, the accumulation of auxin via coordinated cell-to-cell polar transport is indispensable for leaf initiation and determines the sites of leaf initiation (Figure 2C). This is mediated by PIN-FORMED (PIN) proteins, efflux transporters with polar localization (Okada et al., 1991; Bayer et al., 2009). The resulting polarization by PIN1 creates a convergence of auxin flow at the meristem surface, leading to an auxin maximum at defined points (Reinhardt et al., 2003; de Reuille et al., 2006). Through this process, regionalization of the PZ occurs, and initiation of leaf primordia commences.

Central to the initiation and maintenance of the undifferentiated regions of the SAM are class I *KNOTTED-like homeobox* (*KNOX1*) transcription factor genes (Jackson et al., 1994; Long et al., 1996) (Figure 2C). A recent study demonstrated that SHOOT MERISTEMLESS (STM), a KNOX1 protein, physically interacts with WUSCHEL (WUS), a local specifier of stem cell identity (Laux et al., 1996; Mayer et al., 1998; Su et al., 2020), and that this complex enhances WUS binding to the *CLAVATA3* (*CLV3*) promoter in *Arabidopsis thaliana* (Su et al., 2020; Figure 2C). *CLV3* encodes a small peptide that acts as a feedback signal from stem cells to the organizing center, where it delimits *WUS* expression (Fletcher et al., 1999). This feedback limits the number of stem cells (Brand et al., 2000; Schoof et al., 2000). In addition to this function of stem cell maintenance in the SAM, KNOX1 proteins promote cytokinin (CK) biosynthesis (Yanai et al., 2011) and inhibit gibberellic acid (GA) biosynthesis by directly downregulating the GA biosynthesis genes *GA20oxs* in the SAM (Hay et al., 2002). Extensive studies in many plant species have suggested that CK and GA promote cell proliferation and differentiation, respectively (Figure 2D). Hence, the expression of *KNOX1* in the SAM is thought to be indispensable for maintaining an undifferentiated state in this region (Scofield and Murray, 2006).

Auxin maxima created by polar auxin transport exclude the expression of *KNOX1* in the presumptive region of leaf primordia in the SAM, separating the presumptive leaf primordium from the undifferentiated cells. Boundary formation is accompanied by a reduced frequency of cell division and a low growth rate (Kwiatkowska and Dumais, 2003; Barbier de Reuille et al., 2015). *CUP-SHAPED COTYLEDON* genes (*CUC1*, *CUC2*, and *CUC3*) are expressed in the boundary region and are involved in establishing this region (Aida et al., 1997; Vroemen et al., 2003). Additionally, *CUC* genes and *KNOX1* form a positive feedback loop that mutually upregulates each other’s expression (Takada et al., 2001; Blein et al., 2008). As a result, the region where *KNOX1* expression is excluded becomes separated from its expression domain, acquiring the determinate identity of an incipient leaf. This process is regulated by mutual suppression between KNOX1 and ASYMMETRIC LEAVES1 and 2 (AS1 and AS2) proteins, which are involved in the development of flat, symmetric, and extended leaf laminae and their vein systems (Machida et al., 2021; Figure 2C). STM inhibits *AS1* expression in the meristem (Byrne et al., 2000, 2002). *AS1* encodes an MYB domain transcription factor specifically expressed in leaf primordia that interacts with AS2, a LATERAL ORGAN BOUNDARIES DOMAIN protein. Subsequently, the AS1–AS2 complex recruits Polycomb Repressive Complex 2 to stably suppress *KNOX1* expression (Lodha et al., 2013). Conversely, *AS1* in the SAM is also suppressed by KNOX1 (Byrne et al., 2000). Therefore, mutual inhibition plays an important role in forming two distinct domains in the SAM.

The rice (*Oryza sativa*) KNOX1 protein *O.* *sativa* HOMEOBOX1 (OSH1) directly and positively regulates the expression of all five *KNOX1* genes, including *OSH1*, through evolutionarily conserved cis-sequences (Tsuda et al., 2011). This suggests that the positive feedback loop can also facilitate regionalization of the two domains, at least in rice. Additionally, once the two domains are established, the leaf primordia provide feedback to the shoot apex stem cells via auxin transport in the inner cells (Shi et al., 2018). *KNOX2* genes are also known to antagonize *KNOX1* genes to promote leaf development. Ectopic expression of *KNOX2* and *BELL* (BELL-like), its heterodimeric partner and a plant TALE homeobox transcription factor gene, suppresses SAM activity (Furumizu et al., 2015; Figure 2D). Therefore, multilayered regulatory systems seem to be involved in the proper regionalization of the undifferentiated region and presumptive areas of leaf initiation in the SAM.

The leaf primordium, which arises as a bulge, subsequently acquires the adaxial–abaxial (ad–ab) polarity required to generate a flat leaf shape. Microsurgical experiments have demonstrated that separating the incipient leaf primordium from the apical meristem leads to a radial primordium, instead of a normal flat primordium (Sussex, 1951, 1955). Laser ablation analysis of tomato (*Solanum lycopersicum*) confirmed the hypothesis that signals between the meristem and leaf primordium allow for the development of a flattened leaf (Reinhardt et al., 2005). Interestingly, the radialized primordia generated by microsurgical experiments showed abaxialized phenotypes, and laser ablation of the L1 layer was sufficient to disrupt the formation of ad–ab polarity. These results were interpreted to mean that the outermost layer, L1, is required to transmit or perceive the SAM-derived adaxializing signal (Reinhardt et al., 2005).

This signal, known as the “Sussex signal,” is thought to participate in establishing the ad–ab prepattern (Du et al., 2018; Figure 2E). Given that polarity factors, such as class III HD-ZIP transcription factors, have a START domain that is predicted to bind lipophilic ligands (McConnell et al., 2001; Kuhlemeier and Timmermans, 2016), lipophilic molecules are thought to be candidate signals. However, recent studies have suggested that polar auxin transport, not only from the SAM but also from neighboring primordia, is indispensable for establishing ad–ab polarity (Qi et al., 2014; Shi et al., 2017). Additionally, it is unclear whether only the L1 layer in the SAM is involved in this signaling, since the Arabidopsis *pdf2 atml1* double mutant, which lacks epidermal identity, produces flattened leaves with ad–ab polarity. However, abnormalities in leaf blade formation have been observed in this mutant (Abe et al., 2003; Ogawa et al., 2015). One interpretation of these observations is that the epidermis itself is unnecessary for polarity establishment; instead, the outermost layer of cells is sufficient for this process. These findings highlight some major unresolved questions concerning the relationship between the SAM and leaf primordia in establishing ad–ab polarity.

The ad and ab domains of leaf primordia are defined by many factors expressed specifically in the respective domain that mutually interact (Yamaguchi et al., 2012; Du et al., 2018). In this intricate network, class III HD-ZIP transcription factors such as PHABULOSA (PHB), PHAVOLUTA (PHV), and REVOLUTA (REV) specify ad identity (McConnell et al., 2001; Emery et al., 2003). Additionally, class II HD-ZIP proteins such as HAT3 and ATHB4 and the AS1/AS2 complex also determine ad identity (Husbands et al., 2015; Merelo et al., 2016). In contrast, ab identities are determined by the GARP family transcription factors KANADI 1–4 (KAN1–4) (Eshed et al., 2001; Kerstetter et al., 2001) and the auxin response factors ARF2, ARF3/ETTIN, and ARF4 (Pekker et al., 2005; Guan et al., 2017).

Small RNAs also play an important role in establishing polarized gradients of these various proteins. For example, the ad expression of class III HD-ZIP factor genes is restricted by the mobile microRNAs miR165/166. *MIR165/166* genes are expressed, restricting class III HD-ZIP gene expression to the ad domain (Kidner and Martienssen, 2004; Nogueira et al., 2007; Tatematsu et al., 2015; Skopelitis et al., 2017). *MIR165/166* expression is regulated via physical interactions between class III HD-ZIPs and their target genes, class II HD-ZIPs (Merelo et al., 2016). A similar mechanism was observed in the ad domain. Trans-acting siRNAs (ta-siRNAs) produced in the ad domain restrict the expression of *ARF3* and *ARF4* to the ab domain (Chitwood et al., 2009; Schwab et al., 2009; Skopelitis et al., 2017). In addition to the regulation by small RNAs, factors involved in establishing ad–ab polarity are mutually regulated. The AS1–AS2 complex is involved in the epigenetic suppression of *ARF3* and also represses *MIR166* expression (Husbands et al., 2015). In contrast, KAN1 directly suppresses *AS2* expression (Wu et al., 2008; Figure 2E).

Establishing ad–ab polarity is important for leaf blade expansion. In this regard, a study of the snapdragon (*Antirrhinum majus*) mutant *phantastica* (*phan*), later identified as having a mutation at an ortholog of *AS1*, shows a spectrum of phenotypes in leaf lamina expansion. In the most extreme case, leaves are cylindrical and abaxialized, with no lamina expansion (Waites and Hudson, 1995). Similar phenotypes were observed in mutants of other ad–ab polarity-related genes. For instance, *phb1-d* and *KAN1* transactivated by the *AS1* promoter show adaxialized and abaxialized cylindrical leaves, respectively (McConnell and Barton, 1998; Eshed et al., 2001). Additionally, suppression or mutation of genes involved in establishing ad–ab polarity, such as *YABBYs* and *ARFs*, results in defects in the development of leaf lamina (Siegfried et al., 1999; Sarojam et al., 2010; Yifhar et al., 2012). These studies suggest that leaf blade expansion is closely linked to establishing ad–ab polarity.

In their analysis of the *phan* mutant, Waites and Hudson (1995) proposed that lamina outgrowth is promoted at the juxtaposition between ad and ab identities (Figure 2, E and F). Subsequent studies have revealed that establishing the ad–ab domains eventually leads to the establishment of a third region, the middle domain (situated at the juxtaposition between the ab–ad domains) involved in leaf lamina outgrowth. The *WUSCHEL-related homeobox* (*WOX*) genes *WOX1* and *PRESSED FLOWER* (*PRS/WOX3*) play important roles in establishing this middle domain (Nakata et al., 2012). The ad–ab distribution of auxin by ARF activators and repressors is thought to collectively confine *WOX1* and *PRS* expression and the leaf meristematic region to the marginal domain (Qi et al., 2014); subsequently, MONOPTEROS (MP) directly activates the expression of *WOX1* and *PRS* in the middle domain (Guan et al., 2017). YABBY and KAN likely also upregulate and restrict *WOX1* expression, respectively (Nakata et al., 2012). Mutants of the *WOX1* genes in different model species show inhibited lamina outgrowth, suggesting that *WOX1* function in leaf lamina expansion is conserved among species (Vandenbussche et al., 2009; Tadege et al., 2011; Nakata et al., 2012; Du et al., 2020; Zhang et al., 2020; Nakayama et al., 2021). Altogether, these studies suggest that the ad–ab distribution of auxin, ARF activators, and ARF repressors regulates the expression of *WOX1* and *PRS*, leading to leaf expansion and establishing the mediolateral axis (Tadege et al., 2011; Nakata et al., 2012).

Some studies have revealed the roles of mechanical forces in establishing ad–ab polarity. A study on Arabidopsis and tomato suggested that establishing ad–ab polarity leads to mechanical heterogeneity of the cell wall related to the methyl-esterification of cell wall pectins. This heterogeneity appears to produce planar leaf asymmetry (Qi et al., 2017). Furthermore, the cytoskeleton components are primarily aligned with the ab–ad axis, and that this alignment is possibly mediated by a mechanism that senses mechanical stress. This “mechanical feedback” amplifies the initial bilateral asymmetry and promotes directional blade expansion (Zhao et al., 2020). Together, these studies highlight the relatively neglected role of mechanical forces in morphogenesis (Hamant and Traas, 2010). Future work examining establishing ad–ab polarity and the subsequent establishment of the middle domain will need to explicitly address how their underlying gene regulatory networks interact with mechanical signals to allow cell expansion and differentiation to generate a flattened leaf blade that attains its final dimensions.

After gradually losing their proliferative activity, leaf cells enter the second phase of postmitotic cell differentiation. This phase is marked by increased cell size, coinciding with increased vacuole volume and active cell wall synthesis. Studies of Arabidopsis have revealed a relationship between cell proliferation and postmitotic cell expansion at the organ level, suggesting that the connection between the two processes allows the leaf to reach its proper size. However, the exact nature of the relationship between cell size control and final leaf form still remains understudied (see D'Ario and Sablowski (2019)) for the current status of our knowledge of cell size control. The distinct cell proliferation zone coupled with cell differentiation in a developing leaf contributes to leaf shape variation.

Within the context of the developing leaf blade, cell proliferation in the marginal region is gradually suppressed by multiple NGATHA (NGA) and CINCINNATA-class-TCP (CIN-TCP) transcription factors, resulting in determinate leaf growth (Alvarez et al., 2016). Additionally, a recent study demonstrated that CIN-TCP and KNOX2 proteins redundantly suppress cell proliferation activity in the marginal region regulated by *KNOX1* and *CUCs* (Challa et al., 2021). The existence of a marginal meristem restricted to the leaf margin and thought to be involved in leaf lamina expansion, and its contribution to morphogenesis has long been the subject of debate (Maksymowych and Wochok, 1969; Nardmann and Werr, 2013; Tsukaya, 2021). It has been assumed that the factors involved in establishing the middle domain are related to this marginal meristem. However, the precise location of this meristem in leaf primordia and the regulatory mechanisms that establish it are still unclear (reviewed by Tsukaya, (2021)). Leaf morphological diversity is often found in the marginal regions. Therefore, it is essential to understand the relationship between marginal meristematic activity and leaf development, the basic mechanisms of leaf development, and the diversity of leaf morphology.

## Molecular mechanisms underlying leaf form diversification

**Figure 3:**
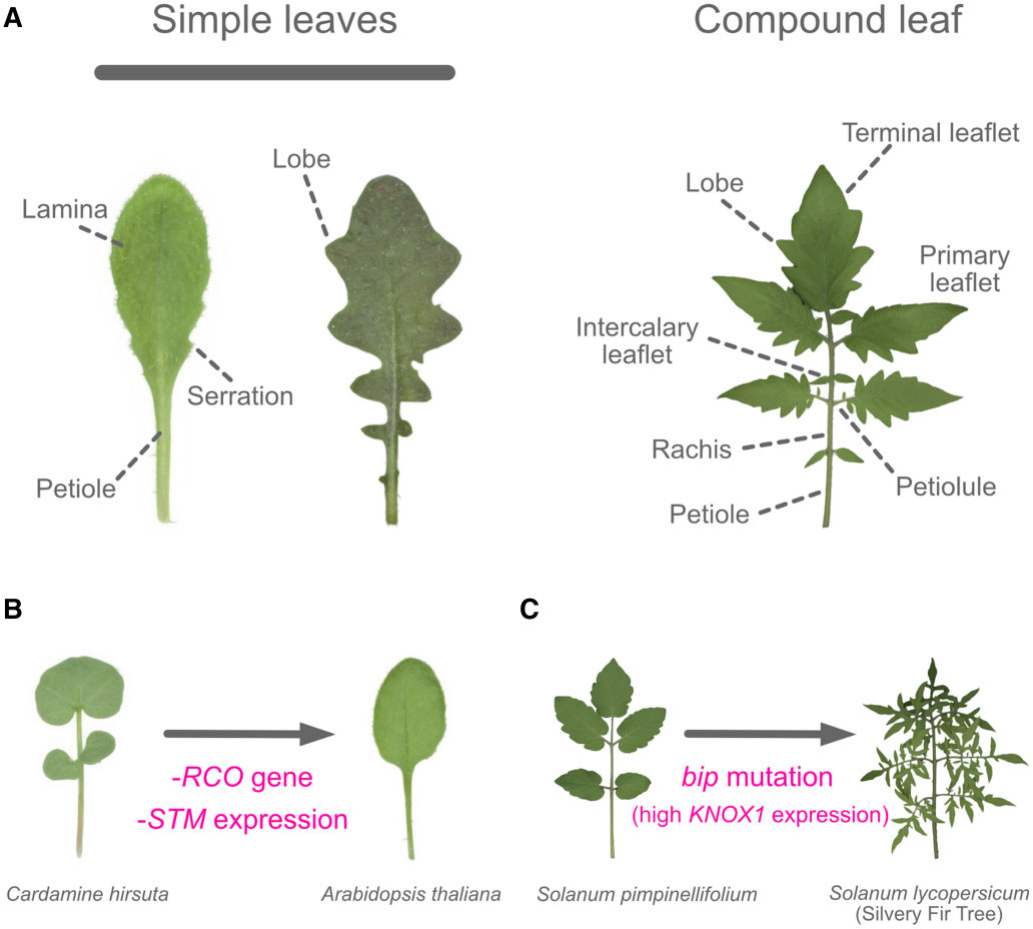
Diagram of simple and compound leaves. A, Diagram of simple and compound leaves, indicating the leaf parts. Left: *Arabidopsis thaliana* (Col-0); middle: *Arabidopsis lyrata* (MN42); right: *Solanum lycopersicum* (M82). Leaf morphologies in the family Brassicaceae (B) and the genus Solanum (C). Note that not all of the above figures are to scale.

The development of model systems with simple leaves is integral to our understanding of leaf development. Species such as *A. thaliana*, *A. majus*, maize (*Zea mays*), and tobacco (*Nicotiana tabacum*) have leaves that are not separated into several independent parts (Figure 3A). This simplicity is an advantage in research on leaf development, and knowledge gleaned from simple-leafed species has been used to understand how more complicated leaf shapes are generated. In nature, a wider range of leaf forms has often evolved in association with different environments, and one major theme in biology is to decipher how developmental mechanisms produce such a variety of morphologies. Over the last two decades, significant progress has been made in identifying and understanding the mechanisms underlying morphological diversity.

*Arabidopsis* *thaliana* has leaves without obvious lobes. This unlobed leaf form is known to be a derived trait (Piazza et al., 2010), and other forms of leaves are observed in the genus Arabidopsis. For instance, *A. lyrata* has lobed leaves (Figure 3A). The evolution of the unlobed leaf in *A. thaliana* involved the loss of *STM* expression in leaves. Although the expression of *KNOX1* genes is involved in the maintenance of the undifferentiated state of the SAM, its expression is suppressed in regions where leaf primordia are initiated (Jackson et al., 1994; Long et al., 1996). Such suppression of *KNOX1* genes is maintained throughout leaf development in simple-leafed *A. thaliana* (Long et al., 1996). Piazza et al. (2010) showed that *STM* is highly expressed in the lobed leaves of some Arabidopsis species, and a selective sweep occurred in the 5′-region of *STM*. These findings raise the possibility that cis-regulatory divergence contributes to the loss of *STM* expression and may become fixed by positive selection (Piazza et al., 2010; Figure 3B).

Moreover, a change in the regulation of *KNOX1* has led to diversification between simple-leafed and compound-leafed Brassicaceae species. *Cardamine hirsuta* (Brassicaceae) has compound leaves: a leaf bearing several individual leaflets borne on a supporting stalk-like structure. A study of *C. hirsuta* showed that *KNOX1* genes are reactivated during leaf development and that their expression is involved in compound leaf development (Hay and Tsiantis, 2006). A *KNOX1* promoter swap experiment between *A. thaliana* and *C. hirsuta* demonstrated that *KNOX1* promoters directed reporter expression in a pattern characteristic of native species, suggesting that differences in the cis-regulation of *KNOX1* genes contribute to species-specific leaf shapes (Hay and Tsiantis, 2006). The K-box is a conserved regulatory element required for *STM* suppression (Uchida et al., 2007). Therefore, changes in K-box sequences might have caused changes in the expression pattern of *KNOX1*, leading to changes in leaf morphology between these species.

REDUCED COMPLEXITY (RCO), a class I HD-ZIP transcription factor, is also involved in promoting leaflet formation in *C. hirsuta* (Vlad et al., 2014). RCO suppresses local leaf growth by regulating the expression of multiple CK-related genes (Vlad et al., 2014; Hajheidari et al., 2019). Studies of *RCO* have revealed the molecular mechanism of compound leaf development and its evolutionary trajectory. *RCO* arose in Brassicaceae through duplication of its ancestral paralog *LATE-MERISTEM IDENTITY1* (*LMI1*), a floral regulator (Saddic et al., 2006), indicating that *RCO* function has been acquired through neo-functionalization. However, *RCO* was secondarily lost in *A. thaliana*, leading to the evolution of a simple leaf phenotype (Figure 3B). Additionally, RCO and ChLMI1 proteins in *C. hirsuta* are functionally equivalent in the developmental context (Vlad et al., 2014). Although it has not received much attention, this observation is consistent with the finding that *A. thaliana lmi1* mutants have an altered leaf phenotype (Saddic et al., 2006). Moreover, a recent study using *Medicago truncatula* showed that both *MtLMI1a* and *MtLMI1b* are required for the proper development of leaf marginal serrations (Wang et al., 2021). These studies revealed the importance of regulatory evolution coupled with gene duplication and loss in generating leaf shape diversity by modifying local growth patterns during organogenesis.

In addition to *C. hirsuta*, the molecular mechanisms of compound leaf development have been studied in tomato and legume species, such as pea (*Pisum sativum*) and *M. truncatula*. Unlike Arabidopsis, *Tomato KNOTTED1* (*Tkn1*), a *KNOX1* ortholog in tomato, is expressed in leaf primordia, and the overexpression of *KNOX1* results in a highly complex leaf phenotype (Hareven et al., 1996; Janssen et al., 1998). The use of different promoters to drive *KNOX* expression indicated that KNOX proteins prolong primary morphogenesis, a stage following leaf initiation, thus allowing leaflet initiation (Shani et al., 2009).

A noteworthy aspect of research on compound leaf development in tomatoes is that the relationship between phytohormones and compound leaf development has been well-studied. *Tkn1* promotes CK biosynthesis and represses GA activity. CK is important for prolonged morphogenesis by promoting cell proliferation, leading to active morphogenesis and a delay in differentiation. CK regulates prolonged morphogenesis at the tomato leaf margin (Shani et al., 2010). Indeed, the manipulation of CK levels led to alterations in leaf complexity (Shani et al., 2010; Shwartz et al., 2016). Meanwhile, GA shortens the morphogenetic window in leaf development by promoting differentiation. *solanifolia* (*sf*), a classic tomato mutant, produces leaves with low complexity and smooth margins. The application of a GA biosynthesis inhibitor suppressed the simple leaf phenotype in *sf*, indicating that elevated GA levels are responsible for the leaf phenotype (Sekhar and Sawhney, 1990, 1991). In *procera*, a DELLA-mutant in tomato with reduced leaf complexity and smooth margins (Bassel et al., 2008; Jasinski et al., 2008; Shwartz et al., 2016), GA application simplified leaf morphology (Shwartz et al., 2016). Additionally, CK and GA exhibit antagonistic activities in various developmental processes (Greenboim-Wainberg et al., 2005). Therefore, hormone-mediated compound leaf development regulated by *KNOX1* appears to play a prominent role in the diversification of tomato leaf morphology.

Based on this framework, Evo-Devo studies have been reported in the section *Lycopersicum* in the genus *Solanum*, which includes cultivated tomatoes and their wild relatives. For example, the Galapagos wild tomato *S. galapagense* shows increased leaf complexity. Kimura et al. showed that *S. galapagense* has a single-nucleotide deletion in the promoter of *PETROSELINUM* (*PTS*), a *KNOX1* gene that lacks a homeodomain. The mutation increases the expression of *PTS*. This alters the interactions of KNOX1 protein with BIPINNATA (BIP), a protein in the BEL1-like homeodomain (BLH) family, because PTS competes with KNOX1 for binding to BIP. Consequently, higher *KNOX1* expression in leaves leads to the increased leaf complexity seen in *S. galapagense* (Kimura et al., 2008). Another example of how changes in KNOX–BIP protein interactions can produce diversity in tomato leaf morphology is provided by Silvery Fir Tree (SiFT), a Russian heirloom tomato showing increased leaf complexity. SiFT has a single-nucleotide deletion in the homeobox motif of the *BIP* gene, leading to a premature stop codon. This truncated BIP protein leads to enhanced expression of *KNOX1* in leaves and a highly complex leaf phenotype (Nakayama et al., 2021). The extreme complexity of the SiFT leaf induced by alteration of the KNOX–BIP interaction likely led to the use of SiFT as an ornamental and landscaping plant (Figure 3C).

Genes other than *KNOX1* have also been reported to function in the evolution of leaf shape diversity. In Fabaceae, compound-leafed species belonging to the inverted repeat-lacking clade (IRLC) use *UNIFOLIATA* (*UNI*), an ortholog of the floral regulator *LEAFY*/*FLORICAULA* (*LFY*/*FLO*), instead of *KNOX1* genes to regulate compound leaf development (Hofer et al., 1997). A recent study demonstrated that PINNATE-LIKE PENTAFOLIATA1 (PINNA1), a BLH protein, and PALMATE-LIKE PENTAFOLIATA1 (PALM1), a C2H2 zinc finger protein, negatively regulate leaf morphogenetic activity by directly repressing the expression of the *LFY* ortholog in *M. truncatula* (He et al., 2020). Therefore, it is important to emphasize that *KNOX1* is not always involved in compound leaf development. The roles of *KNOX* and *LFY*/*FLO* in generating a common phenotype provide an interesting window into evolutionary and developmental mechanisms that require further exploration and are discussed in more detail in a subsequent section.

## Conservation of core networks and finding exceptions to the rules

Despite nearly two decades since their initial identification, the comparative analyses of highly conserved key genes and their core networks continue to be informative starting points for identifying sources of leaf diversity. For many of these genes, their degree of functional conservation has imbued them with a near “natural law-like” status. In particular, two of the most commonly stated “rules” of morphogenetic diversity are that compound leaves result from *KNOX1* reactivation and that leaf blade expansion results from the juxtaposition of polarity factors (e.g. class III HD-ZIPs [C3HDZ] and ARFs). These “rules” provide a framework for hypothesis testing, but we would argue that there remains ample room for exploring their limits. In particular, the continued sampling of nonmodels is leading to a deeper understanding of the morphological limits and mechanistic exceptions to these rules.

**Figure 4:**
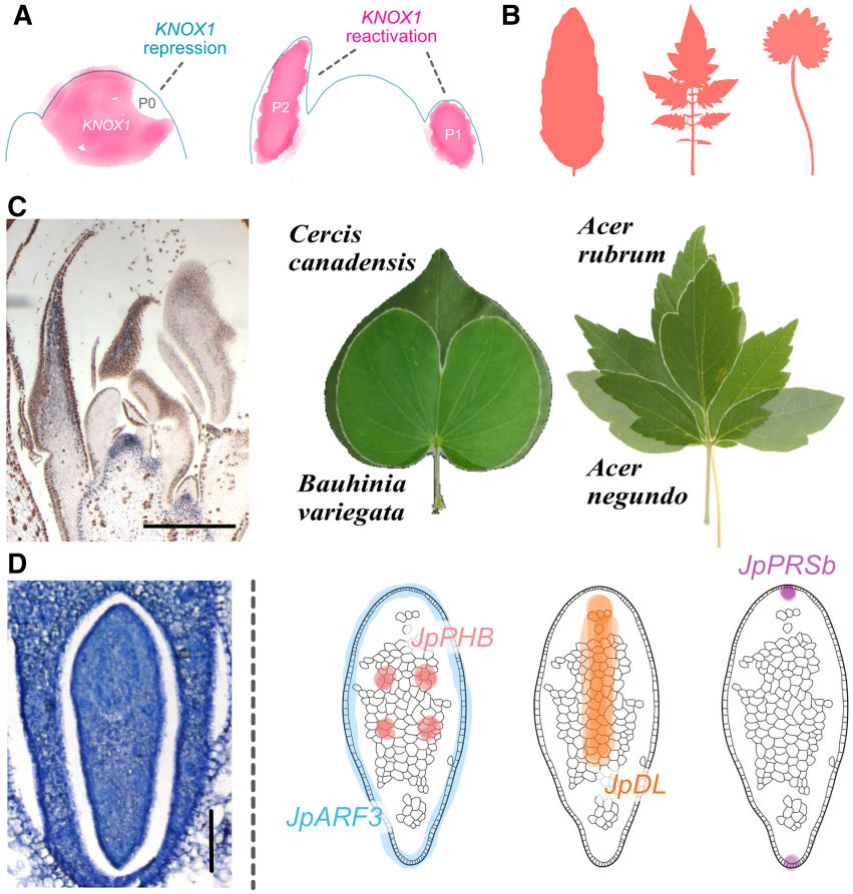
Rules of morphogenetic diversity and their exceptions. A, Image of canonical KNOX1 localization in species with simple leaves (left) or compound or secondarily-simple leaves (right). B, The final leaf forms of “simple” pattern: *Amborella trichopoda* (left); “compound” pattern: *Solanum lycopersicum* (middle); “secondary-simple” pattern: *Pimpinella anisum* (right). C, KNOX1 localization in *Cercis* species. The presence of KNOXI in the simple leaves of *Cercis* species (and the close relationship with *Bauhinia* species with bilobate leaves) supports the notion that these morphologies were derived from an ancestor with compound leaves. Maple species (genus *Acer*) are another example where examining KNOX1 might help resolve whether the majority of species with simple leaves are derived from a compound developmental program. D, Cross section of a unifacial leaf blade from *J. prismatocarpus* and localization of key regulators at an early stage of unifacial leaf morphogenesis. Photograph courtesy of Dr Xiaofeng Yin. Figure modified from Nakayama et al. (2013). Bars = 100 µm.

The reactivation of *KNOX1*-like orthologs in the leaf primordia of species with compound morphology is now understood to be nearly ubiquitous across seed plants. As outlined above, *KNOX1* is repressed in the early initiating leaf primordia and either stays repressed in simple-leaved species such as *A. thaliana* and maize or is reactivated in compound-leaved species such as tomato. However, a number of exceptions to this pattern exist. For example, it appears that some simple-leaved species (e.g. *Lepidium oleraceum*, anise [*Pimpinella anisum*], *Coffea* sp., *Vitis* sp., *Cercis* sp.) have a cryptic compound developmental program early in their initiation (i.e. *KNOX1* is reactivated), which is then secondarily modified to generate a simple morphology (Figure 4, A and B; Bharathan et al., 2002; Champagne et al., 2007). The extent to which diversity in simple leaf morphology is due to the modulation of an ancestral compound in the developmental program remains unknown, but the existing variation in many lineages suggests this mechanism is underappreciated (Figure 4C). Although it seems unlikely, it remains unclear if *KNOX1* expression in such putative secondarily derived simple leaves is functionally vestigial.

Other exceptions to *KNOX1* reactivation have been identified. Members of the IRLC) of legumes with compound leaves do not reactivate *KNOX1* and are instead dependent on *LFY* orthologs for reestablishing an indeterminate state (Hofer et al., 1997; Bharathan et al., 2002; Champagne et al., 2007; Zhou et al., 2014). Interestingly, recent work from legumes outside of the IRLC has revealed that *LFY* is more broadly necessary for generating compound morphology, e.g. in mung bean (*Vigna radiata*; Jiao et al., 2019) and *Lotus japonicus* (Wang et al., 2013b). In fact, in many compound-leaved species (even outside of legumes), such as tomato (Molinero-Rosales et al., 1999), soybean (Champagne et al., 2007), and *C. hirsuta*, (Monniaux et al., 2017), *LFY* often plays a role, albeit a minor one, in generating complexity. At this point, it seems that *KNOX1* and *LFY* often fulfill similar roles in the generation of leaf complexity, yet it remains unclear if their mode of action is the same. Mechanistically, *KNOX1* affects leaf complexity by reactivating a degree of indeterminacy in the leaf (Sinha et al., 1993). Is this also the case for *LFY*, a gene that is sufficient to promote the determinate development of flowers in the meristem (Weigel et al., 1992)? KNOX1 is capable of complementing LFY’s role in leaf complexity in IRLC legumes, suggesting they have similar modes of action. It will be interesting to compare the interacting partners of these two proteins in the leaves of legumes to better understand how *KNOX1* maintains its indeterminate mode of action despite developmental context, while LFY does not.

The other core module of leaf development that rivals *KNOX1* in breadth of analysis is the regulation of leaf polarity and the role that core components like *C3HDZs* play across diverse taxa. The central rule from this work is that juxtaposition of ab–ad polarity is necessary for blade outgrowth—sometimes referred to as the Waites–Hudson model (Waites and Hudson, 1995; Conklin et al., 2019). As outlined above, this process is mediated by a host of polarity factors, which in turn establish the formation of a middle domain where *WUS*-like genes direct cell division (Figure 2, E and F). Although the mechanisms of this rule have been less functionally studied outside of angiosperms, polarity of factors such as *C3HDZs* have been shown to be conserved across ferns and seed plants, but not in lycophytes like *Selaginella* (Floyd et al., 2006; Prigge and Clark, 2006). More recently however, a *C3HDZ* was found to be localized to the ad lamellae cells of gametophyte leaves from the moss *Dawsonia superba* (Yip et al., 2016), making it unclear how complete our picture of *C3HDZ* evolution is without broader sampling of these early diverging lineages.

Within angiosperms, the morphological diversity of leaf types suggests that a dissociation between polarity (or at least canonical factors regulating polarity) and leaf blade expansion may exist. Compared with bifacial leaves, with ab–ad domains, unifacial leaves appear to be derived from a single domain of polarity. In many cases, the leaf is circular and is thus thought to have evolved by radialization from the loss of one domain of polarity. Without the juxtaposition of these domains, the middle domain fails to form, and cell division is mostly uniform in all directions. However, in some unifacial species, a leaf blade is able to form in the ab–ad plane, seemingly defying this rule of leaf morphogenesis (Figure 4D).

Work in the monocot genus *Juncus* has revealed that the unifacially flattened leaves of *J. prismatocarpus* have ab identity. In particular, while a *C3HDZ* gene (*JpPHB*) was adaxially expressed in the bifacial sheath at the leaf base, the gene was confined to vascular tissue in the blade (Yamaguchi et al., 2010). This ab identity was further confirmed by the ubiquitous expression of an *ARF-like* gene in the leaf blade (Figure 4D). Most interestingly, a *WOX* gene (*JpPRSb*) was found to be expressed at the ad and ab tips of the developing blade. Additionally, the mechanism by which *JpPRSb* is polarized is dependent on the *YABBY* gene *DROOPING LEAF* (*DL*), which has a brief period of expression in the midline of P2 primordia (Figure 4D). More recent work has revealed that *JpPRSb* polarization is auxin dependent (Nukazuka et al., 2021), but how *DL* mediates this polarization remains unknown. Of most interest is that the expression pattern of *DL* is associated with thickening cell divisions (division in the ab–ad plane; Yin and Tsukaya, 2019), suggesting an ability to set up a polarity field independent of canonical factors acting in bifacial leaves. It has been proposed that the mechanical feedback mechanism acting in bifacial leaves (discussed above) could also potentially explain lamina growth in the ab–ad plane, but this mechanism still fails to explain how unifacially flattened leaves develop an asymmetry opposite that of bifacial leaves (which is subsequently amplified by mechanical feedback; Jiao et al., 2019; Zhao et al., 2020). Nevertheless, unifacial species represent an interesting model to test the generality of the mechanical feedback mechanism in leaf morphogenesis and require further study.

While it seems plausible that this general mechanism might explain the flattening of unifacial leaves in most monocots, the fact that the *DL* ortholog *CRABS CLAW* does not function in leaf development in dicots (Bowman and Smyth, 1999; Yamaguchi et al., 2004; Fourquin et al., 2005; Ishikawa et al., 2009; Wang et al., 2009; Nakayama et al., 2010) suggests that the independent evolution of unifaciality between these two clades likely occurred by different mechanisms. Given that the diversity of unifacial leaves in eudicots is underappreciated, with almost all work outside of the monocots focusing on species of the genus *Acacia* (Boke, 1940; Kaplan, 1980), multiple unique mechanisms might exist for leaf blade expansion that remain to be identified. In fact, just within the legumes alone, there are a minimum of three independent origins of flattened unifacial leaves. Exploring this diversity will undoubtedly expand our understanding of the rules governing leaf morphogenesis.

## Placing the core networks in their ecological context

Sampling the plant phylogeny more broadly has been a successful means of advancing our understanding of diversification, but it is also increasingly clear that these core networks must be examined in relevant environmental or ecological contexts. One study examined the role of *KNOX1* in a plant’s ability to alter its leaf morphology in response to environmental conditions—a phenomenon known as phenotypic plasticity (West-Eberhard, 2003; Zotz et al., 2011). Phenotypic plasticity leading to changes in leaf form in response to environmental conditions such as light intensity and quality, ambient temperature, and water availability is called heterophylly (reviewed by Nakayama et al., (2017)). Heterophylly is found in many plants, especially aquatic plants, and its molecular mechanism has recently been reported (e.g. Li et al., 2017; Koga et al., 2021). *Rorippa aquatica* (Brassicaceae) is a perennial herbaceous and semiaquatic plant whose habitat includes the shores of lakes, ponds, and streams in North America that exhibits distinct heterophylly between submerged and terrestrial conditions (Nakayama et al., 2014). A study with *R. aquatica* demonstrated that the expression level of a *KNOX1* ortholog is altered in response to changes in the surrounding environment, leading to changes in GA and CK concentrations in leaf primordia. Additionally, exogenous hormone application alters the leaf complexity of *R. aquatica*, providing two different insights worth considering (Nakayama et al., 2014).

The first insight was the discovery of the KNOX-GA-CK module in model plants, which regulates morphological diversification both among species and within a species. A variety of factors have altered the *KNOX1* pathway, including promoter variation, alterations in effective concentrations, and changes in expression patterns, leading to subsequent morphological changes (Hay and Tsiantis, 2006; Kimura et al., 2008; Nakayama et al., 2021). Recent progress in transcriptome analysis and the incorporation of network biology has been helpful in furthering our understanding of morphological regulation. Gene co-expression network (GCN) analysis with three different tomato species revealed that a gene module including *BOP*-*PTS*, which alters the morphology of tomato leaves, is located at the periphery of the gene regulatory network (GRN), while genes that play a more fundamental role, such as those that control cell proliferation, have a more central location in the network. In this GRN, *KNOX1* is thought to serve as a bridge connecting a peripheral gene network module to the core network within the leaf developmental GRN (Ichihashi et al., 2014). This bottleneck location can provoke the rewiring of developmental GRNs, which might explain why the regulation of *KNOX1* was repeatedly manipulated to generate variation in leaf complexity and leaf shape. In other words, *KNOX1* may be a hotspot for morphological evolution, both inter- and intraspecifically, due to its position within the network, where it can easily cause morphological changes. However, GCNs have limitations, as they may not reflect actual protein–protein or protein/DNA interactions. Hence, it will be necessary to integrate gene expression information with data on physical interactions among all factors involved in shoot morphogenesis and to evaluate the location and function of *KNOX1* in the comprehensive network architecture in this context.

The second insight is that the environmental sensitivity of *KNOX1* may be a trigger for morphological evolution. In particular, this sensitivity may allow for evolutionary responses where *KNOXI* plasticity leads in the production of phenotypic variation while genetic fixation follows (Levis and Pfennig, 2016, 2019). This concept, known as genetic accommodation, was first hypothesized by Baldwin (1896) and has since been extensively expounded (West-Eberhard, 2003). This process begins when a novel trait is induced as a result of environmental input (phenotypic accommodation), and genetic fixation by selection (genetic accommodation) occurs when the induction of this trait is reproduced across generations. In fact, there are examples of evolutionary experiments that have reproduced this process. Suzuki and Nijihout explored this phenomenon using larvae of *Manduca sexta* (tobacco hornworms), which change their body color in response to ambient temperature (Suzuki and Nijhout, 2006). The caterpillars emerge green at temperatures above 28°C and black when it is cooler. When selection was applied to the response to heat treatment for 13 generations, a distinct difference in plasticity occurred. That is, strains selected for greater body color change showed a greater degree of change, while strains selected for less change showed no response to heat treatment (Suzuki and Nijhout, 2006). This suggests that in both strains, phenotypic accommodation induced by the environmental factor of heat treatment occurred repeatedly, and genetic accommodation occurred when selection was applied.

The control of leaf complexity involving *KNOX1* is thought to have adaptive significance for the efficiency of gas exchange and for tolerance of low temperatures (Royer et al., 2005). Therefore, it is quite possible that the heterophylly induced by *KNOX1* will be the target of selection and that its fixation will lead to morphological diversification. As mentioned above, molecular mechanisms that regulate heterophylly have now been reported (Nakayama et al., 2014; Li et al., 2017; Koga et al., 2021), and our knowledge of epigenetic mechanisms independent of sequence variation is accumulating as well. These studies demonstrate the utility of examining developmental systems within their ecological context, which will better inform our understanding of the evolutionary events leading to morphological diversification.

## Expanding and moving beyond the core networks

As we have highlighted throughout this review, comparative work on leaf development has been centered on key core networks, but recent advances in genomics now offer the possibility of branching well beyond these core networks. Until recently, attempts to use high-throughput methods to characterize the GRN of leaf development have relied primarily on bulked sequencing of the leaf transcriptomes from different developmental stages (e.g. Wang et al., 2013a; Ichihashi et al., 2014). Further resolution was obtained by bulk sequencing of cell types and domains using laser capture microdissection methods (e.g. Nogueira et al., 2009; Qiao et al., 2020; Martinez et al., 2021). Despite the obvious utility of these methods, the treatment of all cells in a domain, tissue, or stage as homogeneous masks the undoubtedly complex patterns underlying the differentiation and maturation of leaves.

In model systems, pooled sampling strategies that give an averaged read of molecules across all cells are being replaced by single-cell methods. Importantly, these methods often provide not just a view of how cells may differ from their neighbors, but also how they may transition from one state to another in a dynamic fashion, providing insights into the spatio-temporal regulation of development. This feature would be especially important for the SAM and leaf primordia, where compartmentalization and progression of differentiation are occurring within an organ. Cutting edge technologies being developed include spatial transcriptome profiling in situ, single nuclear transcript profiling (snRNA-seq), and simultaneous analysis of DNA accessibility and transcriptome profiles in a single cell (scATAC-seq and scRNA-seq). The challenge to synthesize these data points generated from individual cells into organ and organism-level information has already begun. In fact, scRNA-seq using the SAM of *A. thaliana* showed that cells expressing *STM* (STM^+^) are not undifferentiated uniform cells, but rather cells showing high heterogeneity. Some of the STM^+^ cells can be considered transit-amplifying meristematic cells moving toward leaf identity. This reveals the previously unknown heterogeneity of cells in the SAM and shows the importance of single-cell technology (Zhang et al., 2021).

While protocols are being rapidly developed in several model species (reviewed in Ryu et al., 2021; Seyfferth et al., 2021), the applicability of these methods across the diversity of plant morphologies may require a lot of fine tuning. This is because the generation of single-cell transcriptomes relies on the ability to generate protoplasts. For studies of leaf development, this will often mean generating protoplasts from a shoot apex containing a highly heterogeneous mixture of cell types. Even in model species, this has required significant method optimization to ensure adequate capture of low abundance or delicate cell populations (Satterlee et al., 2020). Many of the nonmodel systems that have been integral to characterizing the core networks of leaf development across the plant phylogeny are also likely to have very different requirements for protoplast generation.

Two alternative methods may offer a more feasible approach for recalcitrant species. The isolation of nuclei for snRNA-seq seems to be more generally applicable across species and tissue types, making it the best choice for studies aimed at comparing single-cell dynamics across species (Sunaga-Franze et al., 2021). One positive aspect of single-cell studies is that by sampling whole apices with leaf primordia of different stages (i.e. a near complete developmental series), little planning is needed to determine how to directly compare developmental stages across species. Alternatively, the generation of spatial transcriptomes is not yet at single-cell resolution (Giacomello, 2021), but this method too seems to be easily adapted across a diverse set of species and tissues (Giacomello et al., 2017; Giacomello and Lundeberg, 2018). This method in particular could be a powerful way of building on the decades of single gene localization studies foundational to our understanding of leaf development. However, unlike snRNA-seq, more thought will need to be given to the collection of different stages when comparative work is being conducted.

## Back to diversity

We are at a turning point in biology. Combined studies of genetics and model organisms have provided a basic framework for development. This has turned our gaze toward understanding diversity, and this research avenue is already providing a treasure trove of information, as described in this review. However, attempts to understand diversity using a basic framework generated from model species raise new questions. For instance, a study in *Sarracenia purpurea* (Sarraceniaceae), a carnivorous plant with pitcher leaves, showed that the tissue-specific direction of cell division, rather than changing leaf polarity, is crucial for the development of pitcher leaves (Fukushima et al., 2015). Furthermore, a recent study of *Utricularia gibba* (Lentibulariaceae), a carnivorous aquatic plant, showed that regional identities modifying growth rates oriented by two orthogonal polarity fields are necessary to provide a more complex leaf shape (Whitewoods et al., 2020). These studies indicate that relatively parsimonious cues can generate diverse leaf forms via a change in the orientation of cell division and/or gene expression involved in the establishment of polarity fields. Therefore, the impact of cell division patterns and mechanical processes in leaf form diversification needs to be clarified.

Finally, regarding GRNs, it is not clear which core modules of leaf development are conserved among angiosperms due to lack of data on many angiosperm clades. Therefore, we are not close to understanding how the astonishing morphological diversity in leaves arose. With new genomes and transcriptomes being assembled rapidly, new methods for naive sampling of cells and tissues or to distinguish different cell and tissue types from a single sample will no doubt be required to help us update the basic framework of leaf development that will be broadly applicable. Beyond this, such studies could provide clues about the astonishing and fascinating diversity in leaf form seen in nature.

## Funding

This work was supported by grants from the United States Department of Agriculture NIFA (2014-67013-21700), National Science Foundation (NSF) IOS (1558900), an NSF Postdoctoral Research Fellowship in Biology (IOS-1812043) and a Katherine Esau Postdoctoral Fellowship to A.R.L., and a Japan Society for the Promotion of Science KAKENHI (JP19K23742, JP20K06682 to H.N.). The laboratory work of H.N. was supported by a Grant-in-Aid for Scientific Research on Innovative Areas (JP19H05670).

*Conflict of interest statement*. None declared.

## Notes

All three authors contributed to writing and approved the final version of the manuscript. N.R.S. integrated all sections of the text. H.N. and A.R.L. created the figures.

The author responsible for distribution of materials integral to the findings presented in this article in accordance with the policy described in the Instructions for Authors (https://academic.oup.com/plcell) is: Neelima R. Sinha (nrsinha@ucdavis.edu).
